# Exercise-Based Interventions for Internet Addiction: Neurobiological and Neuropsychological Evidence

**DOI:** 10.3389/fpsyg.2020.01296

**Published:** 2020-06-25

**Authors:** Shanshan Li, Qianjin Wu, Cheng Tang, Zichao Chen, Li Liu

**Affiliations:** Institute of Sport Science, Sichuan University, Chengdu, China

**Keywords:** exercise-based interventions, internet addiction, neurobiology, neuropsychology, review

## Abstract

With the increase in the number of internet users, the problems associated with excessive internet use have become increasingly obvious. Internet addiction can alter neurobiology, and its symptoms can be alleviated through exercise, but whether exercise exerts these effects through neurobiological pathways is unclear. Here, we reviewed the neurobiological mechanisms of exercise-based interventions against internet addiction by searching PubMed and Google Scholar for relevant research using such keywords as “exercise”, “internet addiction”, “hypothalamic-pituitary-adrenal axis”, “neurotrophin”, and “dopamine”. This review summarizes advances in our understanding of the neurobiological processes through which exercise can reduce internet addiction, and our analysis strengthens the idea that exercise-based interventions can be effective in this regard. The available evidence suggests that exercise can increase the levels of neurotrophic factors, cortisol, and neurotransmitters; improve the morphology of specific parts of the central nervous system, such as by stimulating hippocampal neurogenesis; protect the autonomic nervous system; and control the reward urge. In other words, exercise appears to mitigate internet addiction by regulating the neurobiology of the central and autonomic nervous systems. In this way, exercise-based interventions can be recommended for reducing internet addiction.

## Introduction

The 2019 report of the China Internet Network Information Center showed that the number of Chinese internet users reached 854 million in December 2018, and there are currently 4.5 billion internet users worldwide (CNNIC, 2019; Miniwatts, 2019). While the internet brings convenience and economic benefits to people and organizations, excessive use of the internet can cause addiction, harming their psychological condition and impacting academic, professional and social functions (Beard and Wolf, 2001). Studies in the United States and Europe suggest that prevalence of internet addiction ranges from 1.5 to 8.2%. In Southeast Asia, the prevalence among young people can be as high as 20–30% (Egorov and Grechanyi, 2019). Internet overuse continues to increase due to the low cost, portability, and increasing sophistication of internet-related devices.

Internet addiction is a type of behavioral addiction and is often accompanied by such comorbidities as impulsiveness, depression, anxiety, and obsessive-compulsive disorders (Alimoradi et al., 2019). The behaviors associated with internet addiction include “pathological internet use” (Davis, 2001), “problematic internet use” (Davis et al., 2002), “internet addiction disorder” (Siomos and Angelopoulos, 2008), and “internet gaming addiction” (Freeman, 2008). Internet addiction is currently diagnosed based on criteria analogous to those used to diagnose substance addiction. The physical manifestations are compulsive internet use, and the psychological manifestations are behavioral problems caused by withdrawal from internet addiction (Mihajlov and Vejmelka, 2017). Standard treatments against internet addiction have not yet been established; clinical trials have focused mainly on psychological, pharmacological, and exercise-based therapies. Drug treatment has side effects that may affect mental and physical well-being, while individualized psychological treatment is difficult to design and takes a long time. Therefore, exercise-based interventions may be more practical.

Exercise has been extensively investigated as an alternative or adjunct treatment for internet addiction because it has psychological benefits (Zhang, 2012), such as reducing depression, anxiety, and anger, as well as improves mood (Hassmen et al., 2000). It also has physical benefits, such as strengthening cardiopulmonary function, promoting blood circulation, and improving immune response and nervous system function. A meta-analysis concluded that sports interventions can significantly reduce internet addiction (Liu et al., 2017), and one study showed that exercise can substantially reduce the time spent online and the severity of internet addiction (Kocak, 2018). Therefore, we think that exercise-based interventions may be an effective way to mitigate and even eliminate internet addiction.

## Neurobiological Mechanisms of Internet Addiction

### Internet Addiction and Autonomic Nervous System (ANS) Dysfunction

The neurobiology of internet addiction has attracted much attention, but relatively little is known (Tereshchenko and Kasparov, 2019; Vaccaro and Potenza, 2019). This addiction appears to involve the simultaneous activation of the sympathetic and parasympathetic arms of the autonomic nervous system (ANS). For example, when internet addicts are on-line, their pulse and respiration accelerate, while their peripheral temperature and skin conductivity decrease (Lu et al., 2010). This contradicts an earlier proposal that internet addiction involves antagonism between the sympathetic and parasympathetic systems (Carlson, 2007). The reward-and-aversion hypothesis of addiction may also apply to internet addiction (Huang, 2017): when using the internet, addicts experience a brain reward process; when not using the internet, addicts experience displeasure as a result of withdrawal symptoms (Young, 1996). These mutually reinforcing processes contribute to the development and maintenance of addiction and relapse (Brand et al., 2014). Therefore, interventions designed to create antagonism between sympathetic and parasympathetic nervous systems may be effective at alleviating and preventing internet addiction.

### Internet Addiction and the Hypothalamic-Pituitary-Adrenal (HPA) Axis

The hypothalamic-pituitary-adrenal (HPA) axis is involved in substance addiction and other addictive behaviors (Nawata et al., 2012; Vinson and Brennan, 2013). A study on the activity of the HPA axis in adolescents with internet gaming addiction found that the level of serum cortisol in the addicted group was significantly higher than that in the non-addicted group (*p* < 0.026) (Kim and Kim, 2013). On the other hand, another study found no relationship between the HPA axis and internet use disorder (Geisel et al., 2015). This discrepancy may reflect, at least in part, the relatively small study samples and the possibility that HPA axis dysfunction exceeds the reactive change. It seems plausible, even likely, that the HPA axis is involved in internet addiction because it responds to stress, and stress responses are related to the onset, severity and maintenance of internet addiction (Heinze et al., 2016; Kaess et al., 2017). Early adversity and trauma may also alter the HPA axis to increase risk of internet addiction (McGowan, 2013).

### Internet Addiction and Morphological Changes in the Central Nervous System (CNS)

Numerous imaging modalities have shown that internet addiction is associated with changes in neural structure. Internet addiction has been associated with decreases in the thickness of the left lateral orbit frontal cortex, insular cortex, and entorhinal cortex, as well as with increased thickness of the left anterior central cortex, anterior nerve process, middle frontal cortex, infratemporal cortex, and middle temporal cortex. These changes in cortical thickness are related to control execution, visual image, attention, and memory retrieval functions (Yuan et al., 2013; Zhu et al., 2015). Internet addiction is also related to a decrease in gray matter density in left anterior cingulate cortex, left posterior cingulate cortex, and left island and to a decrease in gray matter volume in bilateral dorsolateral prefrontal cortex, auxiliary motor area, orbit frontal cortex, cerebellum, and left medulla. These areas of gray matter change are related to cognitive control, personality expression and decision-making. Uncontrolled use of internet may be related to the reduction in gray matter volume in prefrontal cortex (Miller and Cohen, 2001; Yuan et al., 2011b; Zhou et al., 2011).

In addition to gray matter abnormalities, internet addiction has been linked to white matter abnormalities, namely an increase in the fractional anisotropy of thalamus, left posterior cingulate cortex and left posterior limb of internal capsule, as well as a decrease in fractional anisotropy of parahippocampal gyrus, prefrontal cortex and anterior cingulate cortex. Indeed, higher fractional anisotropy has been related to addiction and some behavioral disorders (Yuan et al., 2011a; Dong et al., 2012; Lin et al., 2012). In these ways, brain imaging technology, increasingly used to study internet addiction, indicates that the brain structures involved in such addiction are related to reward, decision, memory, and cognitive control.

In addition to these structural changes, internet addiction is associated with functional abnormalities in the brain area. Resting cerebral blood flow in parahippocampal gyrus, amygdala, and insula was significantly higher in addicts than in the control group of one study (Feng et al., 2013), and these areas of altered blood flow are involved in learning and memory. Internet gaming addiction changes the distribution of cerebral blood flow in adolescents (Zhu et al., 2015), but it is not clear whether these changes reflect damage to the nervous system or are secondary changes to compensate for the damage. Internet addiction has been associated with enhanced functional connections between the bilateral cerebellum posterior lobe and middle temporal gyrus but weakened connectivity between the bilateral inferior parietal lobe and right inferior temporal gyrus (Ding et al., 2013). Similarly, internet gaming disorder has been linked to increased regional homogeneity of the inferior parietal lobe, left posterior cerebellum and the left middle frontal gyrus but decreased regional homogeneity of the temporal, occipital, and parietal lobes (Dong et al., 2012). Other works have shown that internet addiction can change the distribution of resting cerebral blood flow, cause dysfunction in connections, decrease the efficiency of inhibition responses, increase brain activity related to game impulses, and decrease brain activity related to game control to an extent that causes control dysfunction (Ko et al., 2009, 2013, 2014).

In addition to functional abnormalities, brains of individuals with internet gaming disorder show reduced metabolism in the anterior cingulate cortex, temporal area, frontal area, parietal lobe and striatum, as well as low metabolic connectivity between the temporal area and marginal area and between the motor area and occipital area (Kim et al., 2019). Areas of decreased metabolism are responsible mainly for the integration of auditory and visual information, as well as physical representation. The change in metabolic connectivity leads to dysfunction of sensory integration and impaired sensory information processing.

In these ways, molecular and functional imaging techniques have shown that internet addiction involves structural changes in brain areas involved in reward, decision-making, memory, and cognitive control. It alters the distribution of resting cerebral blood flow, increases impulsive behavior, and reduces inhibition control and other brain activities. The neurobiology of internet addiction also involves metabolic reduction, and the change in metabolic connectivity causes sensory integration dysfunction.

### Internet Addiction and Neurotrophic Factors

Increasing evidence shows that neurotrophic factors are involved in the regulation of negative emotions and play a key role in treating depression, drug abuse, and other addictions (Levy et al., 2018). A case-control study showed that the levels of glial cell-derived neurotrophic factor (GDNF) in plasma were significantly reduced in internet gaming addicts, and that GDNF level is inversely related to severity of internet addiction and motivation-related cognitive processes (Jeong et al., 2019). GDNF is a neurotrophic factor that has an important role in the maintenance of dopaminergic neurons in several brain regions, as well as in the development, survival, and maintenance of dopaminergic neurons in the midbrain (Carnicella and Ron, 2009). Although the role of GDNF in internet addiction is not clear, GDNF can promote the survival and differentiation of dopaminergic neurons in the midbrain, which is related to the activity of tyrosine hydroxylase in that brain region. These changes may alter synapses and responsiveness of the mid-limbic dopaminergic system, ultimately leading to weakening of stimulation or reward pathways as well as to addiction-related neuroadaptation (Rosenblad et al., 2003). In contrast, another study found that the serum levels of brain-derived neurotrophic factor (BDNF) were not altered in men with internet use disorder (Geisel et al., 2012), so internet addiction may involve different pathophysiology from other types of addiction. Further research is needed to clarify the neurobiological mechanism of internet addiction (Geisel et al., 2013).

### Internet Addiction and Neurotransmitters

In recent years, many studies have shown that internet addiction is related to dysfunction of the dopamine system. One study found that internet addiction reduced the levels of the dopamine D2 receptor and the expression of dopamine transporter, and it dysregulated dopamine D2 receptor, leading to loss of control and forced behavior (Kim et al., 2011; Hou et al., 2012; Tian et al., 2014). Several types of addiction are associated with lower levels of dopamine D2 receptor (Pallanti et al., 2010), indicating that internet addiction may share neurobiological mechanisms with other addictive diseases. Therefore, we speculate that internet addicts may engage more in internet activities in order to obtain more dopamine reward and normalize dopaminergic activity in their brains. The decrease in dopamine transporters may reflect the loss of striatal terminals or the impairment of dopaminergic function in the brain (Hou et al., 2011).

Internet gaming addiction can reduce the plasma catecholamine level in teenagers in the resting state, which manifests as a decrease in adrenaline and noradrenaline levels (Kim et al., 2016). Internet addiction may involve altered autonomic regulation of the central nervous system (CNS), leading to a decrease in catecholamine. This may reduce the responsiveness of internet addicts to external stimulation and lead to cognitive impairment in the long term (Volkow et al., 1996).

### Internet Addiction and Genetic Variation

In recent years, only a small number of studies have examined genetics and internet addiction (Hahn and Spinath, 2017). Gene and environment play a role in internet addiction, leading researchers to investigate twins and their parents. Problematic internet use is heritable, and there are gender differences, with addiction in men showing greater heritability (Li et al., 2014). In the case of addiction to internet gaming, the role of minor alleles is associated with low level of dopamine secretion (in the case of *DRD2* alleles encoding dopamine D2 receptor) and low level of dopamine receptor in prefrontal cortex (in the case of *COMT* alleles encoding catecholamine-*O*-methyltransferase) (Han et al., 2007). Sequencing showed that the T-mutation (CC genotype) in the rs1044396 polymorphism in the *CHRNA4* gene encoding nicotinic acetylcholine receptor subunit alpha 4 was more frequent in internet addicts than in healthy controls (Montag et al., 2012). In addition, there is evidence that internet addiction causes shortening of telomeres and protective structures on the ends of chromosomes (Kim et al., 2019). This shortening may reflect inflammation or oxidative stress. For example, repetitive, long-term activation of the stress response induces the release of proinflammatory catecholamine, which increases cell turnover, promotes oxidative stress, and damages telomeres (Mather et al., 2010; Sofia et al., 2017). Genetic variation may help explain the effects of internet addiction on cognition, emotion and addictive behaviors.

**Figure 1:**
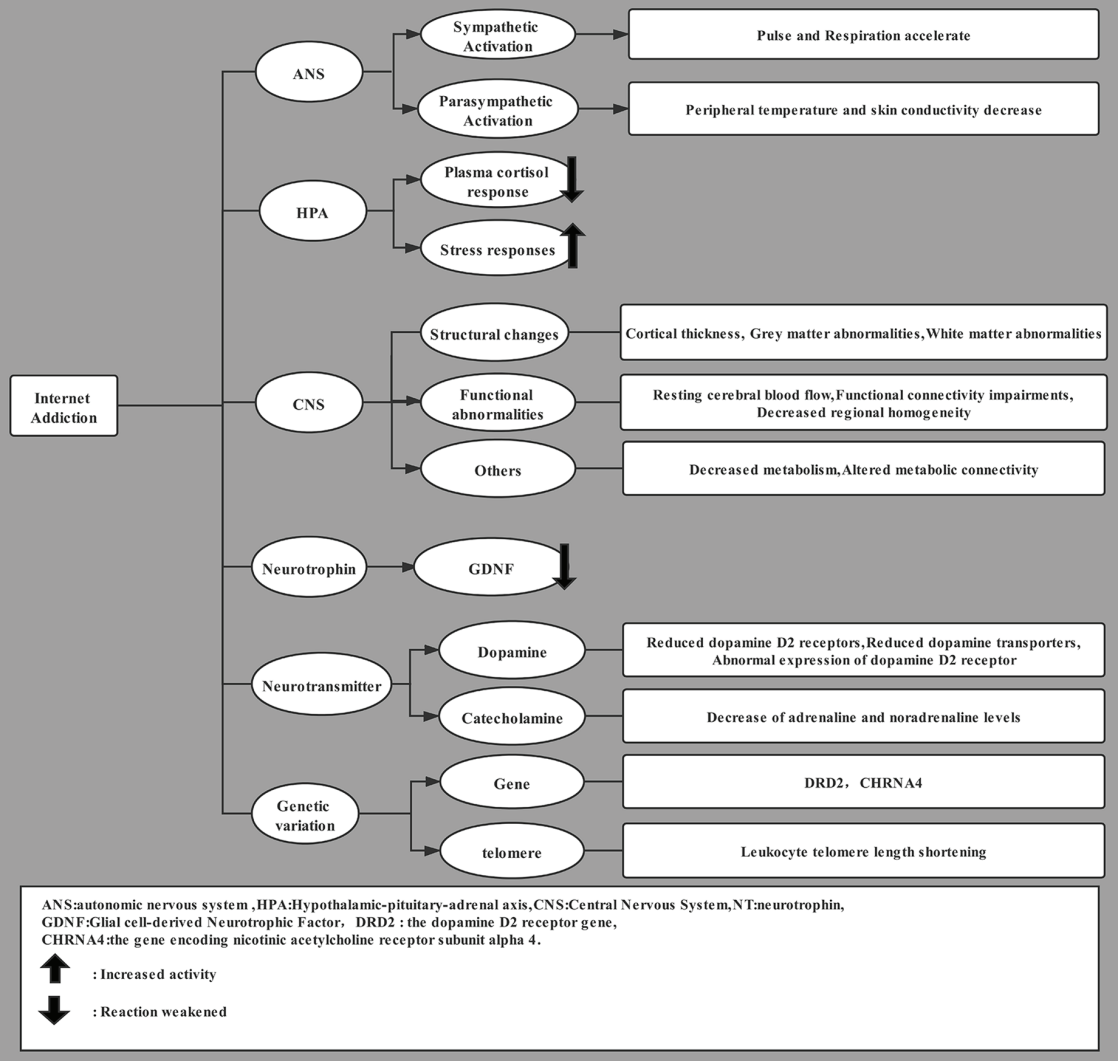
Neurobiological mechanisms of internet addiction.

This literature suggests that internet addiction and substance addiction share the same neurobiological basis (Ko et al., 2009), but there is evidence of differences in BDNF levels between pathological gambling and internet addiction (Geisel et al., 2012). Exercise-based interventions may help counteract the neurobiological causes of internet addiction (Figure 1).

**Figure 2:**
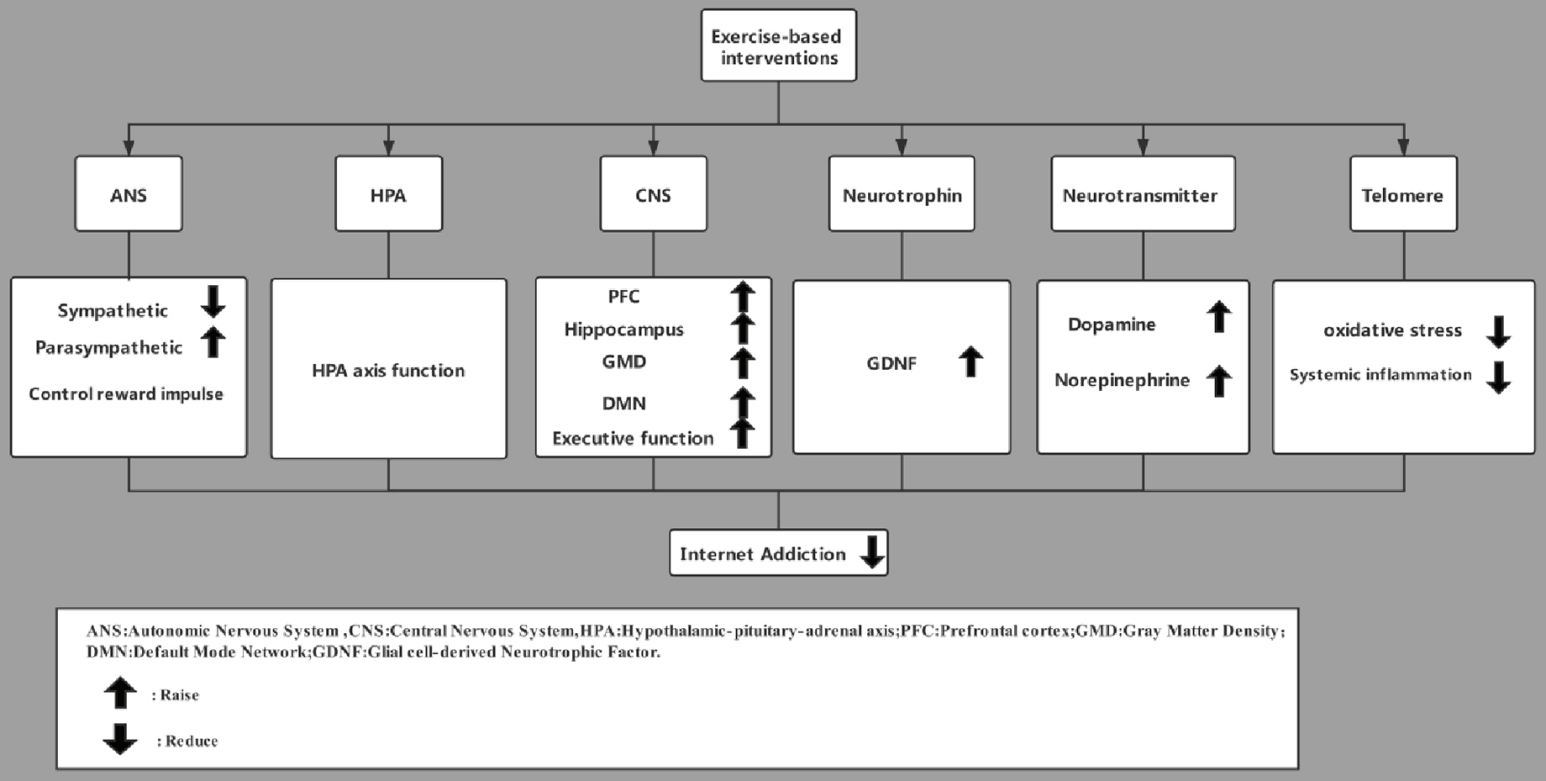
How exercise-based interventions may influence the neurobiology of internet addiction.

## Neurobiological Mechanisms of Exercise Interventions Against Internet Addiction

Exercise-based intervention has been shown to reduce the prevalence and symptoms of internet addiction, and participation in sports can predict internet addiction (Rahimi-Rigi et al., 2019; Hong et al., 2020). In fact, many studies have shown that more generally, exercise-based intervention can be effective against mental diseases (Rosenbaum et al., 2015). The potential explanation for the efficacy of such intervention against internet addiction is that exercise replaces most of the internet experience, as well as improves the physical and mental health of individuals.

### Exercise Improves CNS Structure

Exercise is a natural reward that alters midbrain nigrostriatal dopamine circuits and dopamine circuits involving emotional evaluation (Greenwood, 2019). Animal studies have shown that acute and chronic exercise increase reward-related dopaminergic activity in the striatum circuit (Greenwood et al., 2011; McMorris, 2016), and strenuous exercise causes the human brain to release opioids (Boecker et al., 2008; Saanijoki et al., 2018). In rats, exercise increases expression of the reward-related plasticity marker DFosB in the dorsal striatum and nucleus accumbens (Herrera et al., 2016), and it increases dopaminergic activity in the ventral tegmental area (Dubreucq et al., 2013). These results suggest that exercise can control the reward impulse, which may help explain its ability to mitigate internet addiction.

### Effect of Exercise on the HPA Axis

Exercise affects the HPA axis by reducing the pressure response and producing beneficial effects on health. The difference between the stress caused by exercise and the stress caused by negative life events is that exercise can reverse decreases in cortisol (Heijnen et al., 2016): autonomous exercise can activate the HPA axis and improve cortisol levels (Drogos et al., 2019). Although higher levels of cortisol are associated with cognitive impairment, cortisol responses can be maintained within healthy limits by carefully selecting the exercise type and intensity and by optimizing nutritional status and reducing stress level (Huang et al., 2009; Anderson and Wideman, 2017). For example, more than 12 weeks of intervention based on closed motor skill exercise (e.g., track and field, swimming, and gymnastics) can substantially mitigate internet addiction (Liu et al., 2019).

At the same time, exercise reverses the increase in corticosterone and decrease in glucocorticoid receptor caused by chronic stress, and it induces the HPA axis to adapt to the stress response (Stranahan et al., 2008). Exercise can antagonize the abnormal level of corticosterone in the adrenal gland, hippocampus, and plasma caused by stress stimulation, helping maintain the normal regulatory function of the HPA axis (Heijnen et al., 2016). This dual role of activating and adapting the HPA axis may help explain how exercise can reduce internet addiction.

### Exercise Improves CNS Structure and Connectivity

Long-term exercise promotes prefrontal cortex growth and improves learning, memory, and cognition (Chaddock et al., 2010; Weinstein et al., 2012). A 6-month randomized trial reported a positive correlation between prefrontal cortex volume and exercise (Ruscheweyh et al., 2011). This volume increase may relieve internet addiction as well as concurrent cognitive disorders (Gujral et al., 2017). Similarly, exercise and good fitness level are associated with larger hippocampal volume (Thomas et al., 2016). Moderate-intensity aerobic exercise such as brisk walking for 12 months increased hippocampal volume by about 2% in one study (Inoue et al., 2015). This increase in volume is most obvious in the front part of the hippocampus, which is related to emotion and motivation. Exercise can also serve as an adjuvant therapy to reduce hippocampal apoptosis and oxidative stress-induced neuronal injury (Alipour et al., 2012). In these ways, long-term physical exercise may be effective against internet addiction in part because it increases the volume of hippocampus and prefrontal cortex.

Exercise promotes functional connections between the fronto-parietal and fronto-executive networks of the brain, which are related to the default mode network and cognitive control (Voss et al., 2010a). In a review of nine studies, exercise was found to alter the default mode network (Voss et al., 2010b; Chirles et al., 2017; McGregor et al., 2018). Exercise also alters functional connections in attention, salience, and executive networks (Voss et al., 2019). In this way, improving functional connections among regional networks in the brain may help explain how exercise-based interventions can help alleviate internet addiction.

### Exercise Upregulates Neurotrophic Factors

Animal experiments have shown that exercise affects hippocampus structure and function by regulating levels of neurotrophic factors such as GDNF (Maass et al., 2016; Alves et al., 2019), which plays a key role in the development and maintenance of spinal motor neurons and midbrain dopaminergic neurons (Sopova et al., 2014). In hemi-Parkinsonian mice, exercise increases GDNF levels (Speck et al., 2019). Exercise also improves cardiac function and upregulates neurotrophic factors (Alves et al., 2019). GDNF is involved in the regeneration of damaged axons and regulates neuromuscular connections between synapses in motor neurons (Cortés et al., 2017). By increasing GDNF levels, exercise may normalize axon regeneration in internet addicts, which may help alleviate their symptoms.

### Exercise Upregulates Neurotransmitters

Long-term regular aerobic exercise has a positive effect on monoamine neurotransmitters (Liu et al., 2019). For example, continuous aerobic exercise increases the release of dopamine and norepinephrine in the hypothalamus (Hasegawa et al., 2011), and running increases dopamine level in dorsal striatum (Herrera et al., 2016). Six weeks of treadmill training deregulated adenosine type 1, adenosine type 2A, and dopamine type 2 receptors in rat dorsal and ventral striatum (Clark et al., 2014). Increases in levels of norepinephrine and endorphins may reduce stress and anxiety (Everly and Lating, 2019). Exercise also promotes plasticity in the striatum, which may help reduce internet addiction (Dimsdale and Moss, 1980). The association between low monoamine neurotransmitter levels and negative emotions in internet addiction suggests that exercise may reduce addiction in part by upregulating these neurotransmitters.

### Exercise Preserves Telomeres

A meta-analysis of 19,292 participants showed that telomeres were longer in athletes than in non-exercisers (Lin et al., 2019), and resistance exercise and yoga can stabilize and lengthen telomeres (Krishna et al., 2015). Exercise may exert these effects by increasing antioxidant enzyme activity, making cells more resistant to oxidative stress that would otherwise damage telomeres (Alessio et al., 1988; Radak et al., 2000, 2008). Exercise can help regulate the immune system and improve quality of life (Radak et al., 2008), it reduces the production of adipokines and inflammatory factors produced by adipocytes (Görgens et al., 2015), and it reduces production of inflammatory factors in monocytes and macrophages (Gleeson et al., 2011; Wang et al., 2012). These effects may also help explain how exercise can stabilize and lengthen telomeres.

## Summary and Clinical Implications

Based on neurobiology and neuroimaging technology, internet addiction leads to changes in neural structure, decreases activity of the dopaminergic system and limits neurocognitive function. Exercise-based intervention can effectively improve the ANS of patients with internet addiction and normalize the structure of specific parts of the CNS to some extent. At the same time, it can increase the levels of plasma GDNF and glucocorticoids, the release of neurotransmitters, and the length of telomeres in leukocytes (Figure 2).

The application of exercise-based interventions to internet addiction and other addictions is a rapidly developing scientific field. However, such interventions do carry some risk of inducing exercise addiction. Nevertheless, exercise-based therapy does not have the side effects of drug intervention and other interventions, it is easy to implement, and it can show clinically significant results. Therefore, future research should explore the optimal exercise-based interventions for internet addicts that treat their disease while minimizing risk of exercise addiction. Future work should also examine internet addicts with a broader range of ages and ethnicities, since most studies so far have involved East Asians under 30 years old (Montag and Reuter, 2017). Future studies should carefully differentiate among the many subtypes of internet addiction; explain the observed difference in plasma cortisol levels between those diagnosed with internet gaming addiction or internet use disorder; and explore systematically the differences and similarities between internet addiction and substance addiction, since the two conditions are associated with similar changes in brain structure but different changes in neurotrophic factors. The genetic basis for the observed gender differences in the prevalence of internet addiction should be studied. All these studies can also include a component assessing the safety and efficacy of exercise-based interventions.

## Author Contributions

QW conceived the study. QW and CT searched the literature and selected studies to analyze closely. SL and QW drafted the manuscript. ZC and LL guided the research. All authors revised and approved for publication.

### Conflict of Interest

The authors declare that the research was conducted in the absence of any commercial or financial relationships that could be construed as a potential conflict of interest.
